# Multidimensional Protein Solubility Optimization with an Ultrahigh-Throughput Microfluidic Platform

**DOI:** 10.1021/acs.analchem.2c05495

**Published:** 2023-03-17

**Authors:** Nadia A. Erkamp, Marc Oeller, Tomas Sneideris, Hannes Ausserwoger, Aviad Levin, Timothy J. Welsh, Runzhang Qi, Daoyuan Qian, Nikolai Lorenzen, Hongjia Zhu, Pietro Sormanni, Michele Vendruscolo, Tuomas P.J. Knowles

**Affiliations:** † Yusuf Hamied Department of Chemistry, Centre for Misfolding Diseases, University of Cambridge, Lensfield Road, Cambridge CB2 1EW, U.K.; ‡ Biophysics and Injectable Formulation, Global Research Technology, Novo Nordisk A/S, 2760 Maaloev, Denmark; § Cavendish Laboratory, Department of Physics, University of Cambridge, J J Thomson Ave, Cambridge CB3 0HE, U.K.

## Abstract

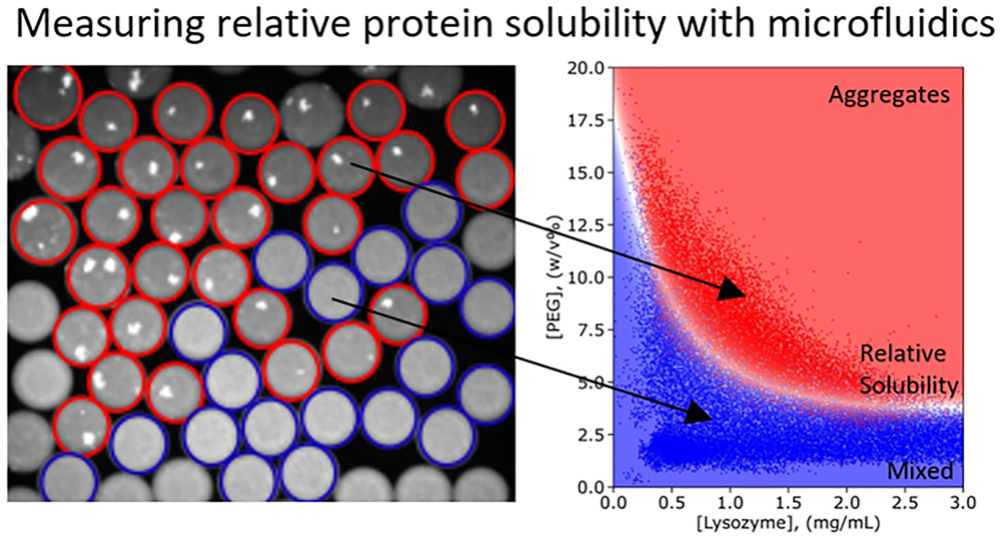

Protein-based biologics are highly suitable for drug development as they exhibit low toxicity and high specificity for their targets. However, for therapeutic applications, biologics must often be formulated to elevated concentrations, making insufficient solubility a critical bottleneck in the drug development pipeline. Here, we report an ultrahigh-throughput microfluidic platform for protein solubility screening. In comparison with previous methods, this microfluidic platform can make, incubate, and measure samples in a few minutes, uses just 20 μg of protein (>10-fold improvement), and yields 10,000 data points (1000-fold improvement). This allows quantitative comparison of formulation excipients, such as sodium chloride, polysorbate, histidine, arginine, and sucrose. Additionally, we can measure how solubility is affected by the combinatorial effect of multiple additives, find a suitable pH for the formulation, and measure the impact of mutations on solubility, thus enabling the screening of large libraries. By reducing material and time costs, this approach makes detailed multidimensional solubility optimization experiments possible, streamlining drug development and increasing our understanding of biotherapeutic solubility and the effects of excipients.

## Introduction

Biologics, such as peptides, proteins, and antibodies, have been the fastest growing drug class over the past decade.^1^ This ascent can be mainly attributed to their inherent low toxicity, high binding affinity and specificity, and favorable pharmacokinetics in comparison to small molecules.^2^ However, many such compounds lack sufficient solubility at the early stages of the drug discovery process, which can result in costly late-stage failures, or yielding products with suboptimal convenience for the patients, e.g., in the form of freeze-dried product that needs to be dissolved prior to administration and intravenous administration instead of subcutaneous administration, which can lead to long hospital visits for each dose instead of home administration.^3−6^ Proteins have evolved to be as soluble as necessary to sustain the required concentrations for their optimal biological functions.^7−9^ The in vivo concentrations of most individual proteins or antibodies, however, are magnitudes below 10–150 mg/mL, which is typical for therapeutic formulations destined for subcutaneous injection.^10^

Solubility measurements remain material- and time-intensive, with the result that they are seldom incorporated at the early stages of drug discovery, where the number of candidates to screen is very high.^11^ The solubility of proteins can be measured *in vitro* using ultrafiltration and ultracentrifugation.^12,13^ Relative solubility measurements have also been developed to rank different proteins or formulation conditions with polyethylene glycol (PEG) or ammonium sulfate precipitation.^14−16^ This reduces material requirements, which is often the primary limitation, and makes it easier to define solubility. Unlike compounds like salt, which in solution are either present as a solid or dissolved, proteins can often populate a plethora of different aggregated states, such as small oligomeric species, amorphous precipitation, and ordered fibrils. This complexity makes the boundary between the soluble and insoluble phase ultimately arbitrary and operationally dependent on the method used to separate the two (e.g., the speed and time of centrifugation).^7^ The relative solubility in these precipitation assays is defined as the amount of PEG or ammonium sulfate that is required for a protein to precipitate out of solution.^12−14^ These methods, however, are relatively low-throughput and still require significant amounts of purified protein material. Moreover, the impacts of formulation parameters, such as pH, ionic strength, and additives, typically used in biotherapeutic formulations are often only assessed by screening each parameter individually while keeping the others constant^17−20^ and often limited to the final stages of preclinical development.^20−22^ It remains highly challenging to carry out multidimensional screenings of formulation parameters, in which solubility is measured by varying two or more parameters together.^20,21,23^ Obtaining solubility data by sampling the multidimensional formulation space as exhaustively as possible is critical as the solubility of biologics is highly formulation-dependent.^20,21,23^ The solubility of proteins can be estimated *in silico* with methods such as CamSol^7,14^ and SAP.^24^ However, such methods cannot be used for the exhaustive screening of formulation conditions, and it remains difficult to base critical decisions solely on computational predictions. Therefore, a new material- and time-efficient experimental method is required to screen candidates at the early stages of development and to optimize protein solubility with respect to various variables, screened individually or in combination.^11^

Here, we present a microfluidic platform to measure the relative solubility of proteins. Previously, microfluidics have been successfully applied to optimize the crystallization of proteins.^25,26^ Here, we demonstrate that protein solubility can be determined at high-throughput at the low cost of 20 μg of purified protein for over 10,000 data points. In comparison with previous methods,^12−14^ this is a 1000-fold increase in datapoints and a greater than 10-fold decrease in required material. This ultrahigh-throughput approach enables optimization of multiple variables in one experiment. In this work, we quantify how effective different formulation additives are at increasing solubility of a protein, measure solubility at different pH values, and select the protein variant with the highest solubility. Thus, this platform has the potential to streamline the drug development process and to greatly increase our understanding of the solubility of biotherapeutics and the effects of excipients.

## Experimental Section

### Materials

PEG (average molecular weight of 6000 g/mol), lysozyme, citric acid, trichloro(1*H*,1*H*,2*H*,2*H*-perfluorooctyl)silane, HEPES, ammonium sulfate, NaCl, sucrose, arginine, histidine, and polysorbate 20 and 80 were obtained from Sigma Aldrich. Alexa Fluor 488 carboxylic acid and Alexa Fluor 647 NHS ester was obtained from Thermo Fisher. BSA was obtained from Fisher Bioreagents and sodium phosphate dibasic from Acros organics. A Sylgard 184 Elastomer base and curing agent were obtained from Dow Corning Corporation. 24 × 60 mm No.1.5 glass slides were obtained from DWK Life Sciences. HFE-7500 was purchased from Fluorochem, and a fluorosurfactant was obtained from RAN Biotechnologies.

### Protein Preparation, Expression, and Purification

BSA and lysozyme were dissolved at 10 mg/mL in 10 mM citrate/phosphate buffer at the desired pH. The proteins were subsequently purified using size exclusion chromatography using a Superdex 75 Increase 10/300 GL for lysozyme and a Superdex 200 Increase 10/300 GL for BSA. The antibodies were kindly provided by Novo Nordisk and were expressed and purified as reported previously.^15^ The sequences and expression and purification procedure are described in detail in the Supporting Information.

### Microfluidic Experiments

**Figure 1:**
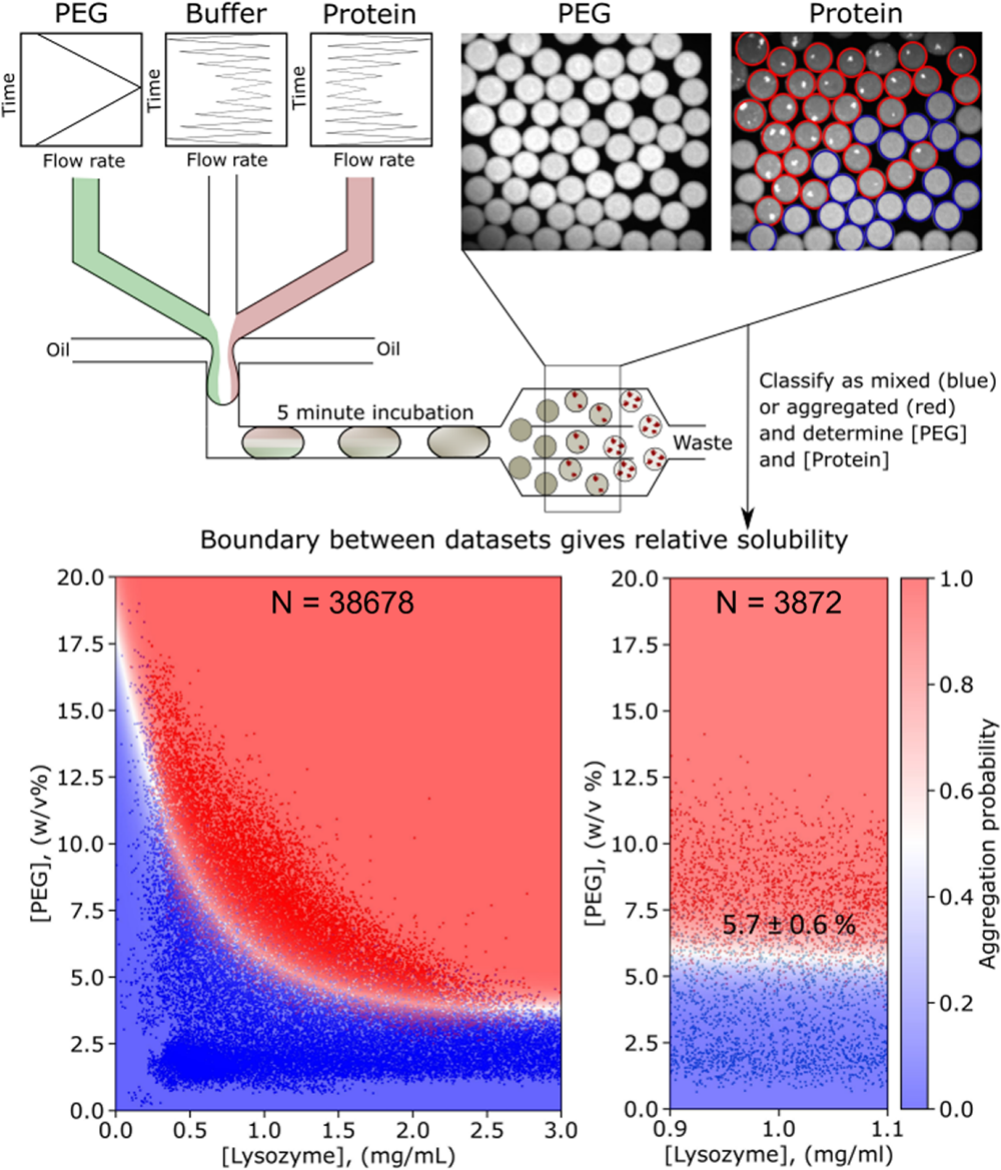
Setup for measuring protein solubility in an ultrahigh-throughput aggregation assay. Water-in-oil droplets of ∼100 pL are created by mixing solutions containing protein, buffer, and PEG at various ratios. The droplets are incubated for 5 min and imaged at the wavelengths corresponding to the fluorescent dyes added solutions. A Python script^28^ is used to determine the concentration of each component and to classify them as containing a mixed solution (blue) or aggregates (red). We fit the data with a support-vector machine algorithm and determine the aggregation probability. This procedure enables us to determine the phase boundary (white region) and the relative solubility value. At 1 mg/mL lysozyme at pH = 7, we find a relative solubility of 5.7 ± 0.6% PEG. The 2D graph on the left and the selection on the right display 38,678 and 3872 data points, respectively.

Standard lithography techniques were used to fabricate microfluidic devices, as shown schematically in Figure 1 (details in the Supporting Information).^27^ For a typical experiment, a solution with just buffer, a solution with protein mixed with 10 molar-% Alexa Fluor 647 NHS ester and left for 15 min in buffer, and a solution with 50 w/v % PEG 6000 in buffer with 2 μM non-reactive Alexa Fluor 488 carboxylic acid would be prepared. Between 5 and 50 μL of each solution is loaded into the tubing (PTFE, 0.012″ ID × 0.030″ OD, Cole-Parmer) connected to the microfluidic device and a gas-tight 0.1 mL syringes (Hamilton 1710) operated by syringe pumps (neMESYS modules, Cetoni). Relative flow rates are varied, while the total aqueous flow rate is a constant value between 60 and 150 μL/h. HFE-7500 oil with 1.5% fluorosurfactant (RAN biotechnologies) is pushed using a gastight 1 mL syringe (Hamilton 1710) into the microfluidic device at a constant rate, between 50 and 200 μL/h, such that droplets of around 100 pL in volume at around 100 droplets/s are obtained. Droplets travel through the microfluidic device for 5 min (or a different time for Figure S1) before being imaged at all relevant wavelengths, typically at 488, 546, and 647 nm. Each set of images contains around 100 droplets and thus 100 datapoints. For accurate intensity to concentration conversion, we obtain three sets of images (*n* = 10 for each condition): images of droplets containing only one of the solutions (calibration), images of a homogeneous solution of all dyes (illumination differences of the lasers), and an image without any sample (background noise of a camera).

### Image Analysis

The images of the droplets were processed by a previously published Python script^28^ that analyses microfluidic droplets, obtains concentrations based on intensities, and looks for inhomogeneous intensities within droplets, which is how aggregates are detected. Further analysis of pictures was performed using Fiji. Figure 4a was created using Chimera.

### Data Fitting

The phase boundary between mixed and aggregate-containing droplet populations was determined by fitting the data using a support-vector machine algorithm with linear or 2nd degree polynomial kernel, programmed in Python.

## Results and Discussion

### Measuring the Relative Solubility of Biologics Using Microfluidics

We have capitalized on advancements in microfluidic technology^28,29^ to generate thousands of microdroplets containing protein, additives, and precipitants (Figure 1, top). Each droplet gives an individual data point where the protein is in a different environment. Through modulating the flow rates of solutions containing protein, buffer, PEG, and selected excipients, droplets with an array of compositions are created. A fluorescent dye, either free in solution acting as a barcode or protein-bound, is added to the solutions. After incubating the droplets for 5 min, they are imaged at the wavelengths corresponding to the added dyes. The concentrations of the compounds in the droplet can then be inferred from fluorescence intensity. Moreover, as the protein is fluorescently tagged, we can directly observe if it is found homogenously in the droplet, or if it has formed precipitates, which appear as bright specs in the droplets. Droplet detection and analysis of their contents are performed using previously developed Python-based image analysis software.^28^ Two groups of data are plotted, with blue datapoints corresponding to mixed samples and red datapoints indicating samples with aggregates. By fitting data using a support-vector machine algorithm, we can determine precipitation probability under different conditions. The probability from 0 to 1 is then visualized by a color gradient layered on top of the data (continuous legend). We can construct phase diagrams in 2D (Figure 1, bottom left) or determine the boundary at a certain protein concentration (Figure 1, bottom right). The white area shows the phase boundary between the mixed phase and aggregated phase, and it is predicted based on the data. The thickness of this boundary indicates how accurately we have measured it and gives the standard deviation of the measurement. For example, at 1 mg/mL lysozyme, the relative solubility of lysozyme is 5.7 ± 0.6% PEG (Figure 1, bottom right), consistent with previous measurements performed with a standard PEG-precipitation assay.^23^

Using the microfluidic platform, the average volume per sample is just 100 pL. Droplets are created at a rate of 100 droplets/s, and thus thousands of datapoints can be prepared in very minimal time. Due to the small size of samples, the incubation time is also greatly reduced, from hours or days^12,14^ to 5 min. We found that varying the incubation period from 2 min to 3 h yielded no significant difference in the observed relative solubility, suggesting that our measurements are performed at near-equilibrium conditions (Figure S1). Additionally, this method provides highly reproducible measurements (Figure S2). Instead of PEG, ammonium sulfate is also often used to measure relative solubility. Ammonium sulfate can similarly be used in our setup for relative solubility measurements (Figure S3). Using this data, classical solubility curves can also be made, with the calculated aggregation probability plotted against PEG concentration (Figure S4).

### Optimization of Formulations

**Figure 2:**
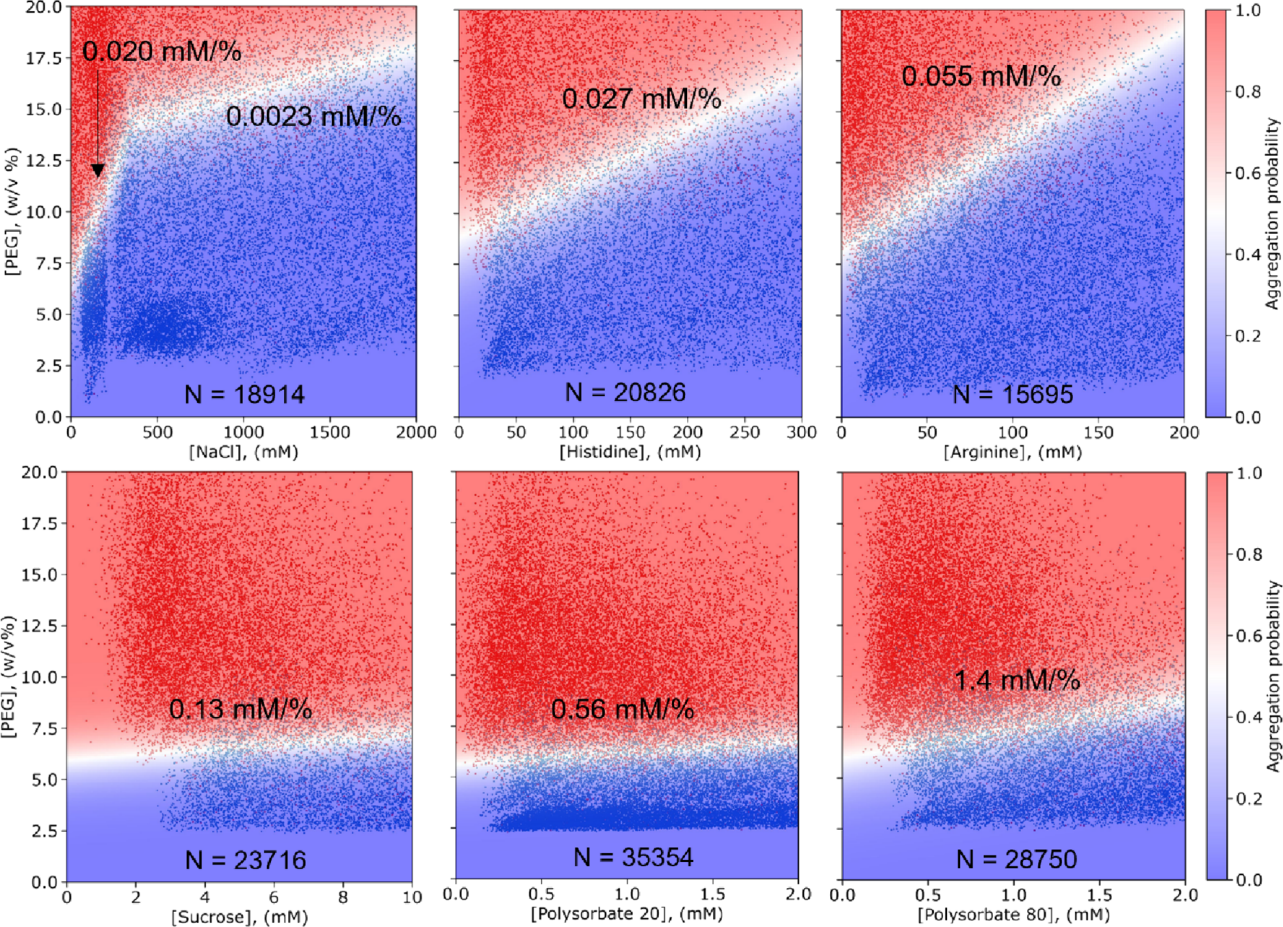
Formulation optimization screens with commonly used additives. Various amounts of additives are added to a 1 mg/mL BSA solution at pH = 5, increasing the relative solubility from 7.3 ± 0.7 w/v % PEG linearly in most cases (Figure S2). The slope of the white boundary, shown in the graphs, states how much %PEG the relative solubility is increased per mM of additive. Thus, the ability of formulation additives to improve the solubility can be compared quantitatively. Of the six compounds tested, the surfactants polysorbate 20 and 80 most effectively improve solubility. The amounts of data points shown are 18,914, 20,826, 15,695, 23,716, 35,354, and 28,750 for the graph of NaCl, histidine, arginine, sucrose, polysorbate 20, and polysorbate 80, respectively.

To develop a protein drug, it is important to identify a formulation that enables a long shelf life.^30^ Here, we have selected common excipients used in therapeutic formulations: sodium chloride (NaCl), histidine, arginine, sucrose, and polysorbate 20 and 80.^31^ These compounds are commonly added as stabilizers to improve physical stability (arginine and NaCl), buffer the pH (histidine), provide an ionic osmotic pressure adjuster (NaCl), and provide a non-ionic osmotic pressure adjuster (sucrose) and as surfactants that prevent interaction of the drug with interphases (polysorbate).^31^ In the absence of these compounds, the relative solubility of BSA at pH = 5 is 7.3 ± 0.7 w/v % PEG (Figure S2). The addition of these excipients can significantly increase the relative solubility to up to 16 w/v% PEG (Figure 2). Moreover, with the microfluidic platform, we can quantitatively compare the ability of the additives to improve protein solubility. We find that, within the explored concentration range, the additives improve the solubility linearly, as would be expected in an ideal solution.^32^ By comparing the slope of the white boundary, displayed in the graphs, we can rank these compounds according to their ability to increase protein solubility. Notably, while small amounts of NaCl improve the solubility at 0.020 PEG%/mM, adding additional NaCl beyond 350 mM only increases the solubility by 0.0023 PEG%/mM. Initially, in the salting-in regime, more salt interacts with the charges on the protein, reducing the interprotein interactions and thus the propensity to form aggregates. However, adding further amounts of salt will not influence the solubility much or could even harm the solubility in the salting-out regime.^16^ Using the measurements in figure 2, we can rationally design a formulation to improve the solubility of the protein under scrutiny. For example, the most suitable surfactant to add to the formulation would be polysorbate 80 as it improves the solubility by 1.4% PEG per mM, rather than polysorbate 20, which improves it by only 0.56% per mM polysorbate. To improve the physical stability of the protein, we would prefer to add arginine over NaCl since arginine improves the solubility more per mM. At 0.13% per mM, sucrose can also significantly improve solubility. Last, if histidine is added to buffer the formulation, we can expect a minor improvement to the solubility. Instead of comparing the slopes of the curves, one can also quantitatively compare the excipients using the area under the curves.

### Directly Comparing Excipients

**Figure 3:**
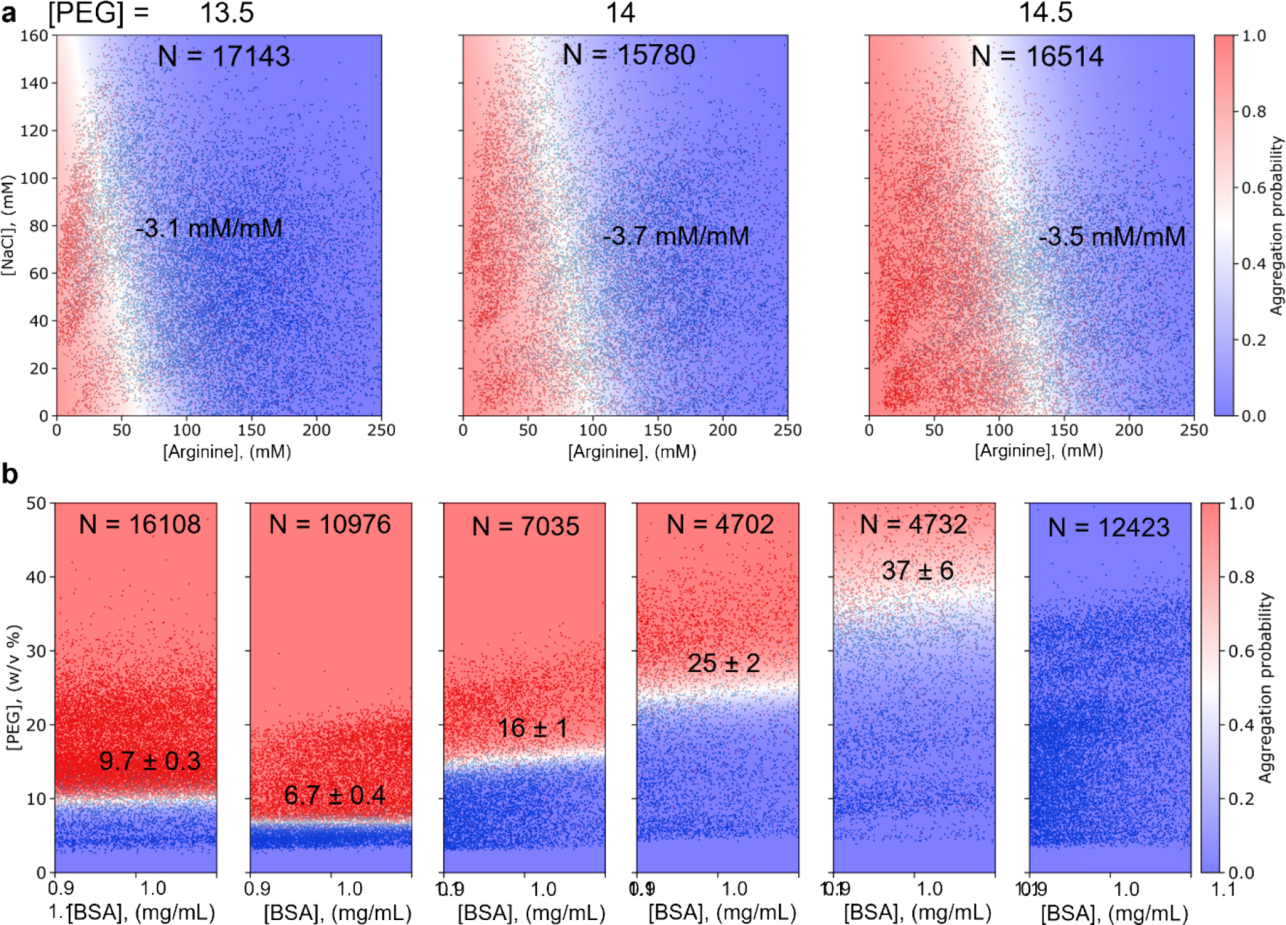
Comparison of formulation additives and optimization of the pH. (a) In the absence of NaCl and arginine, 1 mg/mL BSA at pH = 5 forms aggregates at PEG concentrations of 13.5, 14, and 14.5 w/v%. However, by adding NaCl and arginine, homogeneous solutions are obtained. The linear boundary shows that NaCl and arginine works together additively or linearly to increase the solubility. Based on the boundary slope, we can also conclude that arginine is more effective than NaCl at increasing the solubility. Adding more PEG decreases the solubility linearly since it shifts the boundary to the right. From left to right, the amounts of data points shown are 17,143, 15,780, and 16,514. The slope, NaCl/arginine, is shown in the graph. (b) Relative solubility measurements of BSA at pH = 4, 5, 6, 7, 8, and 9, showing that this protein has the lowest solubility around pH = 5 and a higher solubility at a pH below or above 5. From left to right, the amounts of data points shown are 16,108, 10,976, 7035, 4702, 4732, and 12,423.

Using this platform, we can also investigate combinatorial effects of different additives with high resolution in one experiment, saving additional time and material when improving the formulation. We varied the concentrations of two excipients, which improve the physical stability of the protein, NaCl, and arginine, while keeping the amount of protein and PEG constant (Figure 3a). We obtain aggregates (red) in the absence of NaCl and arginine (bottom left corner) since we have 1 mg/mL BSA and PEG = 13.5, 14, or 14.5% in this experiment. By adding NaCl or arginine, we can improve the solubility and obtain a mixed solution (blue). From the shape of the slope, which is linear, we can also conclude that NaCl and arginine work together linearly to improve the solubility. Since the slope is negative, we can see that both compounds improve the solubility. If the value is −1, the compounds are equally well capable of improving the solubility. A value lower than −1 or higher than −1 indicates the compounds on the *x*-axis or *y*-axis, respectively, is more efficient at improving the solubility of the protein. Here, we find that the slope is around −3.4 and thus that the compound on the *x*-axis, arginine, is more effective at improving the solubility. This matches with our finding in Figure 2, where arginine improved the solubility by 0.055%/mM and NaCl at these concentrations by 0.020%/mM. Three diagrams were produced at PEG = 13.5, 14, and 14.5%. We observe that the boundary shifts linearly to the right and that more NaCl and/or arginine are required to obtain a mixed solution. This is consistent with the fact that PEG promotes aggregation.

### Optimizing the pH of a Formulation

pH is another important factor in protein solubility that can be scanned with the microfluidic platform. We measured the solubility of BSA in a formulation buffered at pH = 4, 5, 6, 7, 8, and 9 (Figure 3b). BSA has a relative solubility of 9.7 ± 0.3% PEG at pH = 4 and a solubility of 6.7 ± 0.4% at pH = 5. Thus, in a formulation, if only solubility was considered, pH = 4 would be more suitable. Alternatively, increasing the pH can improve the solubility significantly, with the solubility at pH = 9 being so high that the boundary could not be determined under these conditions. The measured values and trend compare well with the pI of approximately 4.9^33^ and to previously reported measurements carried out with standard PEG-precipitation assays (Figure S5).^23^ Notably, in comparison to the previous measurements,^23^ the microfluidic-based measurements shown in Figure 3 require 90% less of the protein, have an incubation time of 5 min instead of 2 days, and have a smaller measurement error as they comprise thousands of data points instead of just tens.

### Comparison of the Solubility of Different Mutational Variants

**Figure 4:**
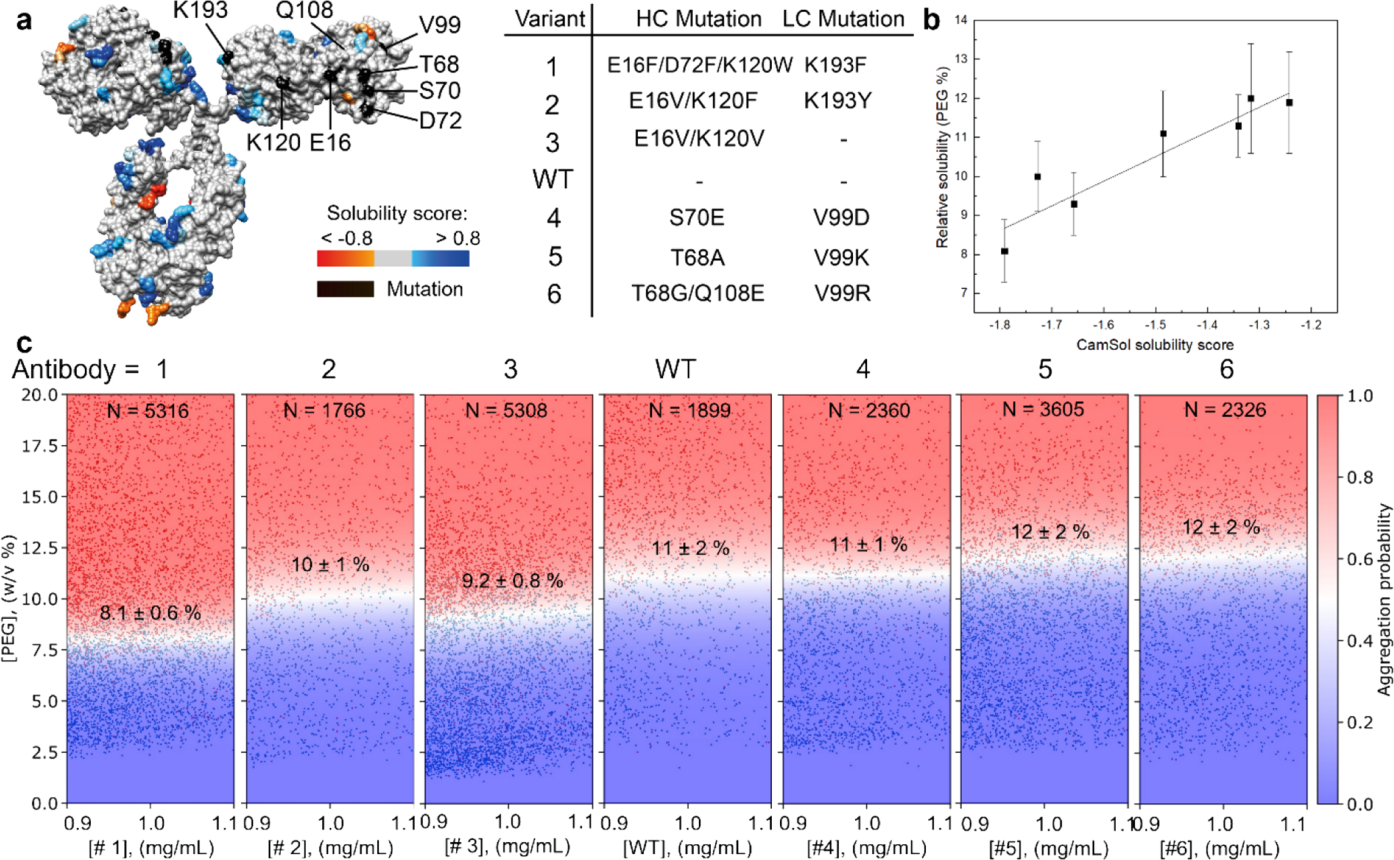
Solubility measurements of an IgG4 antibody and six mutational variants. (a) Structure of the IgG4 antibody, with the solubility score of regions and locations of mutations depicted on the surface. Six variants are used containing two to four point mutations. (b) The relative solubility of the six variants and the wildtype is measured by our microfluidic platform and plotted against the CamSol solubility score.^15^ A clear trend in solubility between these proteins can be measured, despite the small difference in sequence. *r*^2^ = 0.86. (c) Microdroplet-based relative solubility measurements of the variants and wildtype. From left to right, the diagrams contain 5316, 1733, 5308, 1899, 2360, 3605, and 2326 data points.

Another way to improve protein solubility is to mutate the protein to identify more soluble variants while maintaining its desired function.^7,14^ Moreover, screening campaigns of biologics typically yield in the range of tens to thousands of candidates that may differ by as little as one mutation.^2,34,35^ Our microfluidic platform can be employed to measure the solubility of protein mutants. Figure 4a shows the structure of IgG4 antibody, of which the wildtype and six of its previously designed mutational variants were used.^15^ The CamSol method was used to estimate the relative solubility of these variants,^15^ showing that variants 1–3 are expected to be less soluble than the wildtype, while variants 4–6 are expected to be more soluble (Figure 4b). Figure 4c shows the solubility measurements of the mutants and wildtype containing over 1500 data points each. The wildtype has a relative solubility of 11 ± 1% PEG. The variants 1–3 indeed have a lower relative solubility and variants 4–6 a higher solubility. Comparing these measured values with the prediction by CamSol,^15^ we observe that the variants with the lowest CamSol score, meaning they are predicted to aggregate the easiest, indeed require the least amount of PEG to form aggregates. The results also matched the solubility trend observed previously in a 6 week experiment where the antibodies were incubated at elevated temperatures (Figure S6).^18^ Even if these variants differed only by two to four point mutations in the context of a full IgG of about 1350 residues, the microfluidic platform allows us to select variants 4–6 as having a higher solubility than variants 1–3 and thus allows us to optimize the protein sequence.

## Conclusions

Optimizing protein solubility remains a significant challenge in the development of protein-based drugs. High solubility is required for long-term storage and to ensure efficient administration. However, available techniques have high material requirements and not enough throughput to measure protein solubility during early development or to optimize the formulation extensively. We have presented a microfluidic platform that addresses both concerns. We can quantify the solubility increase obtained by different kinds and different concentrations of formulation additives, can optimize the pH, and can study how the additives influence solubility in each other’s presence. The relative solubility can be determined with either PEG or ammonium sulfate, both industry standards. Additionally, our method enables the accurate solubility ranking of protein variants, even when these differ only by a few point mutations. Our microfluidic platform provides a highly quantitative strategy for improving a wide range of aspects influencing protein solubility and can aid in the development of new protein-based drugs.

## Supporting Information Available

Relative solubility measurements, robustness of the protein solubility measurement, comparison of measurements carried out on the microfluidic platform and on a plate reader, comparison of antibody solubility measurements, antibody expression and purification, and fabrication of microfluidic devices (PDF)
