# Supplementary material for: Multidimensional Protein Solubility Optimization with an Ultrahigh-Throughput Microfluidic Platform
Source: Anal Chem. 2023 Mar 17;95(12):5362–8. doi: 10.1021/acs.analchem.2c05495 (PMC10061369; doi:10.1021/acs.analchem.2c05495)
Supplement: Supplementary file 1 — ac2c05495_si_001.pdf [file ac2c05495_si_001.pdf]

Supporting information

Multi-dimensional protein solubility optimization with an ultra-high-throughput microfluidic platform

Nadia A. Erkamp[1], Marc Oeller[1], Tomas Sneideris[1], Hannes Ausserwoger[1], Aviad Levin[1], Timothy J. Welsh[1], Runzhang Qi[1], Daoyuan Qian[1], Nikolai Lorenzen[2], Hongjia Zhu[1], Pietro Sormanni[1], Michele Vendruscolo[1], Tuomas P.J. Knowles[1,3*]

[1]*Yusuf Hamied Department of Chemistry, Centre for Misfolding Diseases, University of Cambridge, Lensfield Road, Cambridge, CB2 1EW, United Kingdom.*

[2]*Biophysics and Injectable Formulation, Global Research Technology, Novo Nordisk A/S, 2760 Maaloev, Denmark*

[3]*Cavendish Laboratory, Department of Physics, University of Cambridge, J J Thomson Ave, Cambridge, CB3 0HE, United Kingdom*

**Email: tpjk2@cam.ac.uk*

Supporting information contains:

- Supporting figures 1-6
- Supporting experimental section
- Supporting references

Supporting figures

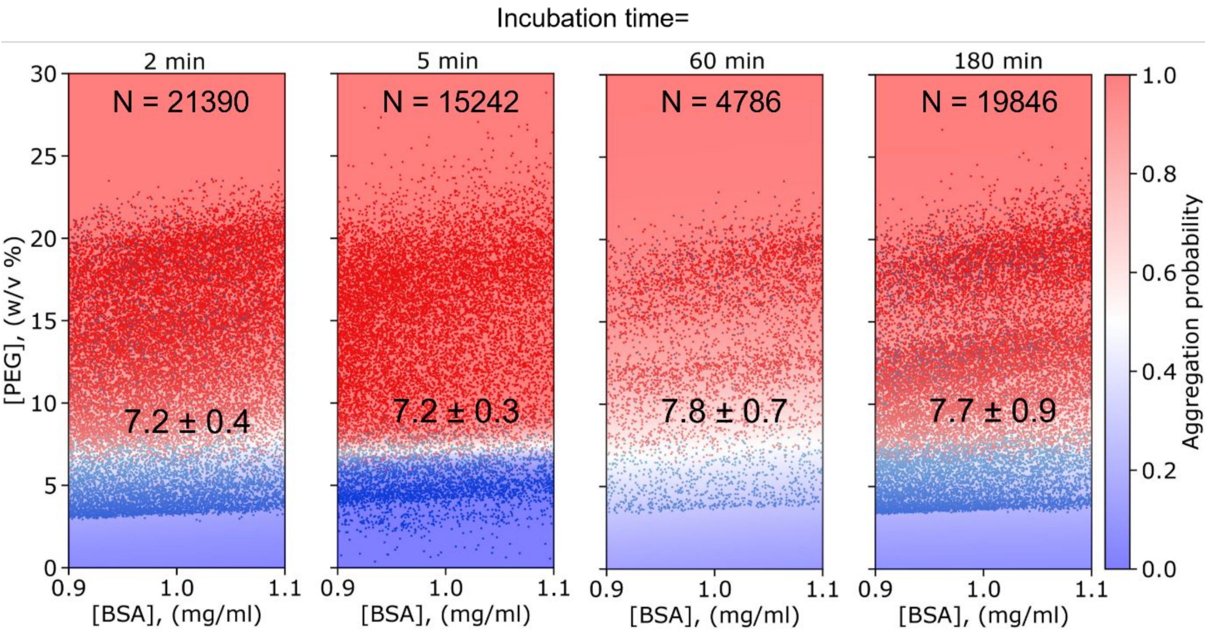

Figure S1. Relative solubility measurements after different incubation times. To assess the effects of different incubation times, we measure the relative BSA solubility at pH = 5 following microfluidic droplets incubation for 2, 5, 60 and 180 min. Notably, while previous work required incubation times of up to 48 hours,[1] our smaller samples are mixed and can aggregate in much less time. All other data in this paper were acquired after a 5 min incubation period. From left to right, the graphs contain 21390, 15242, 4786 and 19846 data points.

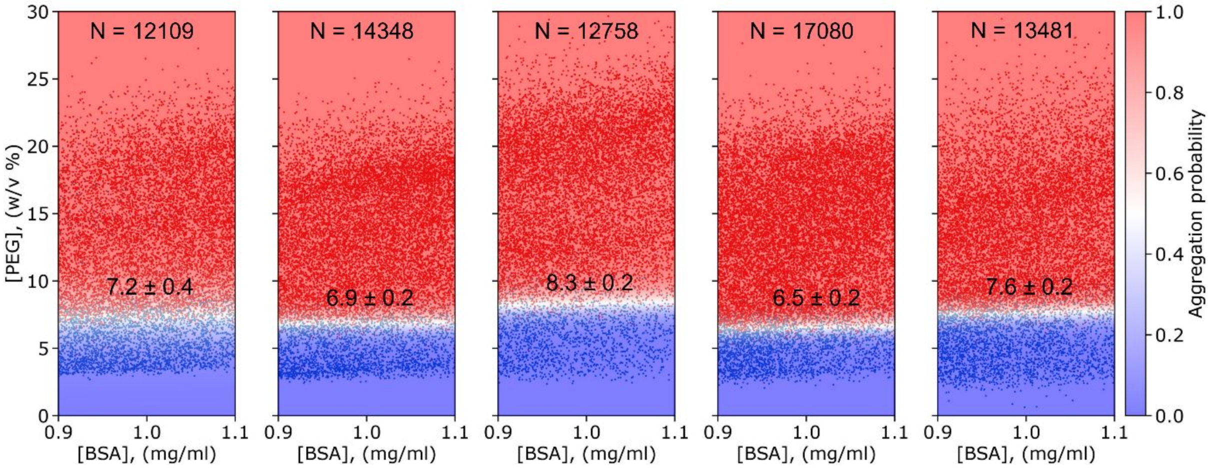

Figure S2. Robustness of the protein solubility measurement. Relative solubility of BSA batches at pH = 5 was measured in different microfluidic devices on different days. The results are highly reproducible. Combining these 5 measurements, we also find that the relatively solubility of BSA at pH = 5 is 7.3 ± 0.7 w/v % PEG. From left to right, the graphs contain 12109, 14348, 12758, 17080 and 13481 data points.

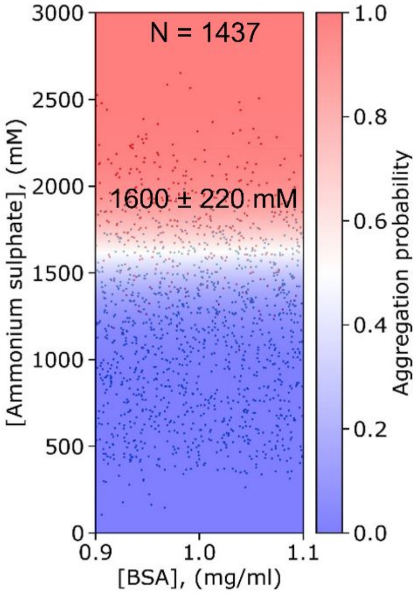

Figure S3. Relative solubility measurement of BSA with ammonium sulphate. Both PEG and ammonium sulphate[2] are industry standards to determine the relative protein solubility. Here, we measure the relative BSA solubility at pH = 5 using ammonium sulphate, instead of PEG and find a relative solubility of 1600 ± 220 mM ammonium sulphate. 1437 data points are shown.

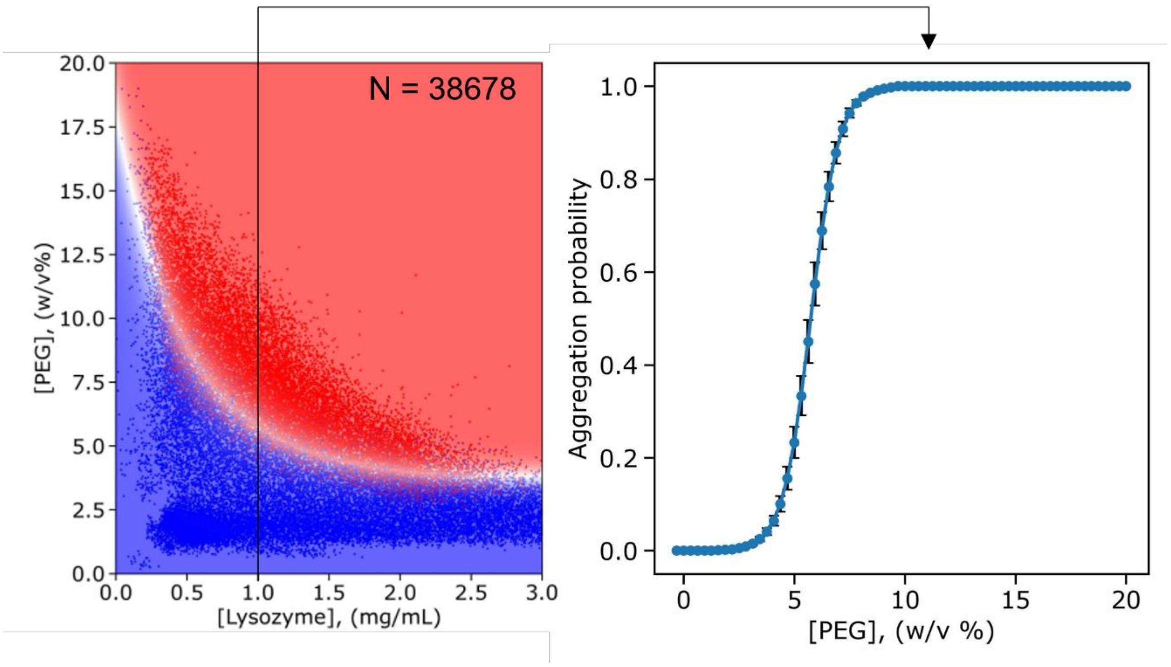

Figure S4. Relative solubility measurement data plotted as classical solubility graph. From the data acquired, the aggregation probability is calculated using a support-vector machine algorithm. At a constant protein concentration, we can also plot this probability as a function of PEG concentration, resembling a classical solubility graph. As an example, this is shown for the lysozyme data from figure 1 at 1 mg/mL.

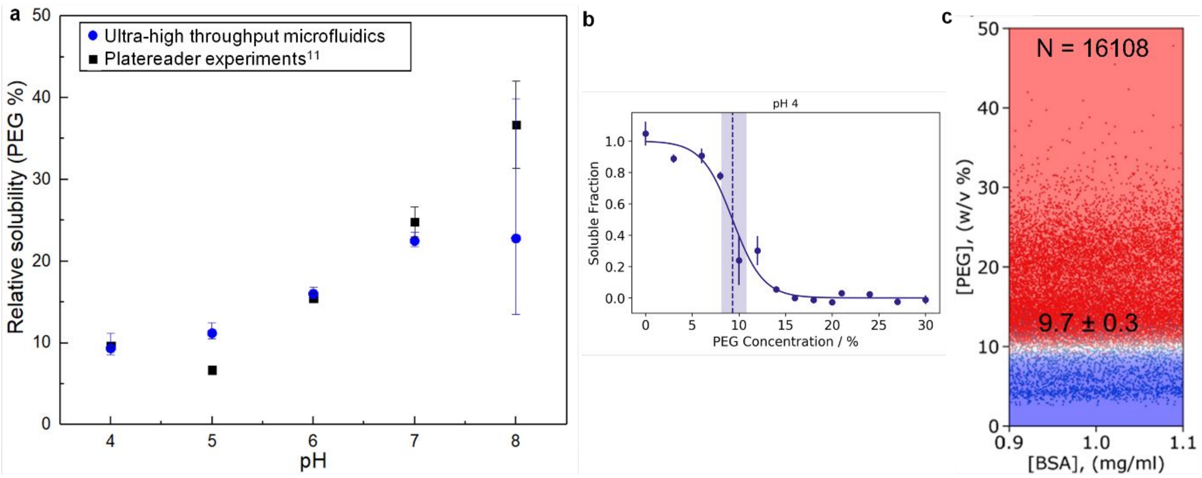

Figure S5. Comparison of measurements carried out on the microfluidic platform and on a plate reader. (a) The relative BSA solubility at pH = 4, 5, 6, 7 and 8 is measured using out ultra-high-throughput microfluidic platform and previously determined using a platereader.[3] We see that we obtain a very similar trend and values. Notably, in comparison with the plate reader experiment, the microfluidic platform has an incubation time of 5 minutes instead of 48 hours, uses only 10% of the protein, results in a smaller measurement error, gives thousands instead of 15 data points and can screen for another variable, like protein concentration, at the same time. Values from **Figure 3** and a previous publication.[3] **(b)** Example of measuring relative solubility using a plate reader.[1] Briefly, BSA at pH = 4 is mixed to obtain a final

concentration of 1 mg/mL with 15 different amounts of PEG using a pipetting robot and incubated at 4 °C. After 48 hours, the plate is centrifuged and the supernatant is transferred into a fresh plate. The amount of BSA that is dissolved in solution is compared with the total amount to give the soluble fraction. Via fitting, the amount of PEG at which 50% of the protein is in solution is determined, which is used as the relative solubility. The relative solubility was found to be 9.3 with an asymmetric error from 8.1 to 10.8.[3] **(c)** Measurement of BSA solubility at pH =4 using the ultra-high-throughput microfluidic setup, see also **Figure 3b**. The relative solubility was found to be 9.7 ± 0.3 using 16108 data points.

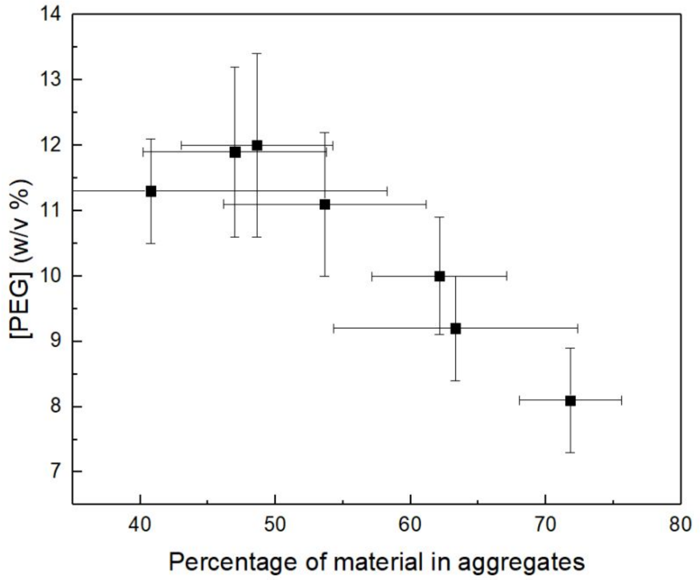

Figure S6. Comparison of antibody solubility measurements. The relative solubility of the antibodies was measured using the microfluidic platform and previously in a 6-week long experiment. Previously, the antibodies were incubated at 45 °C for 6 weeks, after which the amount of material in aggregates was assessed. [4] Antibodies which aggregate at a lower amount of PEG have more material aggregated after 6 weeks, meaning the same trend is observed. Notably, the microfluidic platform has a sample making, incubation and measurement time of minutes and uses over ten times less material, making it an attractive alternative.

Supporting Experimental section

Antibody expression and purification

WT Heavy Chain sequence:

QVQLQESGPGLVKPSETLSLTCTVSGGSISSGYWNWIRQPPGKGLEWIGTISYSGDTY

YNPSLKSRVTISVDTSKNQFSLKLSSVTAADTAVYYCARYGSYVFDYWGQGTTVTV

SSASTKGPSVFPLAPCSRSTSESTAALGCLVKDYFPEPVTVSWNSGALTSGVHTFPAV

LQSSGLYSLSSVVTVPSSSLGTKTYTCNVDHKPSNTKVDKRVESKYGPPCPPCPAPEF

LGGPSVFLFPPKPKDTLMISRTPEVTCVVVDVSQEDPEVQFNWYVDGVEVHNAKTK

PREEQFNSTYRVVSVLTVLHQDWLNGKEYKCKVSNKGLPSSIEKTISKAKGQPREPQ

VYTLPPSQEEMTKNQVSLTCLVKGFYPSDIAVEWESNGQPENNYKTTPPVLDSDGSF FLYSRLTVDKSRWQEGNVFSCSVMHEALHNHYTQKSLSLSL

WT Light Chain sequence:

DIVMTQTPLSLSVTPGQPASISCRSSQSLLHSNGNTYLHWYLQKPGQSPQLLIYKVSN RFSGVPDRFSGSGSGTDFTLKISRVEAEDVGVYYCSQSTHVPWTFGQGTKVEIKRTV AAPSVFIFPPSDEQLKSGTASVVCLLNNFYPREAKVQWKVDNALQSGNSQESVTEQD SKDSTYSLSSTLTLSKADYEKHKVYACEVTHQGLSSPVTKSFNRGEC

The expression and purification of the antibodies was kindly performed by Novo Nordisk as reported previously[5]. Briefly, vectors with mutations were produced using pNNC340 (expression vector harbouring WT HzANTP heavy chain) and pNNC341 (expression vector harbouring WT HzANTP light chain) using QuickChange Lightning multi-site-directed and sitedirected mutagenesis kits, respectively (Agilent Technologies). Strings with the mutations (Thermo Fisher Scientific) with 15 basepair long overhangs on both sides were added to the PCR mix (KOD xtreme kit, Merck Millipore) with pNNC340-41 as template (InFusion HD cloning kit (Takara Bio). The material was transformed into E. coli DH5alpha competent cells (Thermo Scientific), and plated on LuriaBertani (LB) agar plates with carbencillin and incubated at 37°C overnight. Single colonies were inoculated in 2 mL of LB medium with carbenicillin and grown at 250 rpm shaking at 37°C overnight. A QIAGEN Plasmid Plus 96 BioRobot Kit (Qiagen) and a Biomek FXP pipetting robot (Beckman Coulter, Brea, US) was used to harvest the plasmids and the sequence was confirmed Eurofins Scientific's sequencing service. Clones harbouring the correct vectors were similarly to before re-transformed, plated, and single colonies were grown. Using GenElute HP maxiprep kit (Sigma-Aldrich), over 1 mg of each vector was obtained and transfected in a 1:1 ratio for the heavy and light chain into Expi293F™ cells (Thermo Fisher Scientific, Waltman, US) with a density of 3∂106 cells mL−1 and over 95% viability (NC-3000 NucleoCounter (Chemometec). Cultures were grown at 36.5°C, 8% CO_2 and 125 rpm shaking. After 5 days, the cultures were harvested. The supernatant was filtered and the amount of antibody was quantified by Dip and ReadTM Protein A (ProA) Biosensors in an Octet system (Pal ForteBio). The protein was purified on an Äkta Express chromatography system using affinity purification using Mabselect Sure Protein A resin and then Superdex200 resin (GE Healthcare). The column was washed with 0.1 M HEPES pH 7.4, 150 mM NaCl, and antibodies were eluted with 0.1 M sodium formate, pH 3.5 into a pre-equilibrated gel filtration column, with a running buffer of 20 mM HEPES, 0.15 M NaCl, pH 7.4. Eluted fractions were collected in a 96-well plate. Fractions were pooled to obtain high purities and minimum higher molecular weight protein. The concentration of the antibody was determined by absorbance at 280 nm absorbance with Dropsense 96 (Trinean).

Fabrication of microfluidic devices

Microfluidic devices were prepared using standard lithography techniques[6]. Briefly, the device was designed in AutoCAD (AutoDesk) and printed on a photomask (Micro Lithography). UV expose was used to place the pattern of the mask on a silicon wafer coated with a 50 μm thick

layer of SU8-3050 photoresist (Microchem), after heating at 95 °C for 45 minutes. Excessive SU-8 photoresist was removed using propylene glycol methyl ether acetate (Sigma), after heating 5 minutes at 95 °C. The wafer with SU-8 patterns, or master, was dried and served as a mould to make PDMS, poly(dimethylsiloxane), devices. PDMS base and curing agent were mixed in a ratio of 10:1 and baked for 1 hour at 65 °C. The PDMS was then removed from the mould and cleaned by sonication in ethanol. Holes were punched where liquids were put into or would go out of the device with a biopsy puncher. Both the PDMS and a glass slide were activated in an oxygen plasma oven (30 s, 40% power, Femto, Diener Electronics) before bonding together. The device was heated at 95 °C for 3 minutes. The channels were treated with 1% (v/v) trichloro(1H,1H,2H,2H-perfluorooctyl)silane in HFE-7500 (Fluorochem) for 1 minute and subsequently dried with nitrogen gas and then heated at 95 °C for 10 minutes.

Supporting References

1. Oeller, M., Sormanni, P. & Vendruscolo, M. An open-source automated PEG precipitation assay to measure the relative solubility of proteins with low material requirement. *Sci. Rep.* **11**, (2021).
2. Duong-Ly, K. C. & Gabelli, S. B. Salting out of proteins using ammonium sulfate precipitation. in *Methods in Enzymology* **541**, (2014).
3. Oeller, M., Sormanni, P. & Vendruscolo, M. Sequence-based prediction and measurement of pH-dependent protein solubility. *Biophys. J.* **121**, (2022).
4. Kopp, M. R. G. *et al.* An accelerated surface-mediated stress assay of antibody instability for developability studies. *MAbs* **12**, (2020).
5. Wolf Pérez, A. M. *et al.* In vitro and in silico assessment of the developability of a designed monoclonal antibody library. *MAbs* **11**, (2019).
6. Mazutis, L. *et al.* Single-Cell Analysis and Sorting Using Droplet-Based Microfluidics. *Nat. Protoc.* **8**, 870–891 (2013).
